# Chinese undergraduates' mental health help-seeking behavior: the health belief model

**DOI:** 10.3389/fpsyg.2024.1377669

**Published:** 2024-05-16

**Authors:** Haojing Wang, Zhuowen Feng, Zitong Zheng, Jiachen Yang

**Affiliations:** College of Literature and News Communication, Guangdong Ocean University, Zhanjiang, Guangdong, China

**Keywords:** undergraduates, mental health, help-seeking behavior, health belief model, online questionnaire

## Abstract

The detection rate of mental health problems among undergraduates has recently risen significantly. However, undergraduates underutilize mental health services; approximately a third only of undergraduates in need of treatment use school counseling resources. Based on a social psychological theoretical framework, the health belief model, factors of undergraduates' willingness to seek help when dealing with psychological problems were investigated. A cross-sectional online questionnaire and a snowball sampling method with 446 undergraduates investigated perceived susceptibility, perceived severity, perceived behavioral benefits, perceived barriers, self-efficacy, and cues to action to understand how students' mental health-seeking behaviors are affected. We found that perceived susceptibility (*p* < 0.01), perceived severity (*p* < 0.01), perceived benefits (*p* < 0.01), perceived barriers (*p* < 0.01), self-efficacy (*p* < 0.01), and cues to action (*p* < 0.01) significantly correlated with behavioral intention. Encouragement or counseling from others would be more likely to motivate undergraduates to seek mental health help. In addition, we used a bias-corrected Bootstrap approach to test the significance of the mediating effect, the mediation effect of cues to action between undergraduates' perceived susceptibility and mental health help-seeking behavior was utterly significant [mediation effect value of 0.077, with an SE value of 0.027 and a 95% CI (0.028, 0.133)]. It demonstrated that those who perceived themselves to be at high risk of developing a mental illness and who had received encouragement or counseling to seek mental health help were more likely to be motivated to seek mental health help. Multiple regression analyses indicated that self-efficacy (*Z* = 5.425, *p* < 0.01) and cues to action (*Z* = 6.673, *p* < 0.01) independently influenced behavioral intentions. Encouragement or counseling from others would be more likely to motivate undergraduates to seek mental health help.

## 1 Introduction

The frequent occurrence of undergraduates' mental health problems has received increasing attention from society. A meta-analysis conducted in Mainland China found that the detection rates of anxiety, depression, sleep problems, and suicide attempts among undergraduates have increased significantly in the past 10 years (2010–2020), with the depression detection rate being 20.8% and the anxiety detection rate being 13.7% (Chen Y. et al., 2022). The probability of developing physical symptoms of psychological disorders while suffering from depression and anxiety is as high as 25% (McNealy and Lombardero, 2020); more serious cases such as self-harm, suicide, and other life-threatening issues also exist. The most commonly diagnosed conditions within a suicidal group were depression (40%), schizophrenia (7%), and alcohol dependence (7%), and only 7% of them had ever seen a mental health professional (Phillips et al., 2002).

Despite the significant increase in the detection rate of major psychological problems among undergraduates in the last decade, this group has not fully utilized mental health services. Although many schools have set up relevant mental health resources, only about one-third of college students needing treatment will take the initiative to utilize them (Downs and Eisenberg, 2012). Many studies have investigated the psychological help-seeking behavior of university students and found that university students in psychological distress do not choose to seek assistance actively. Yu's study of Chinese university students showed that 1/4 would adopt negative coping strategies. If they decided to seek help, university students preferred to seek help from friends, followed by lovers and family members, and less often chose counselors. Many studies have investigated the psychological help-seeking behavior of undergraduates and found that undergraduates in psychological distress do not actively choose to seek assistance. Yu's study showed that a quarter of Chinese college students would adopt negative coping strategies. If they decided to seek help, college students preferred to seek help from friends, followed by lovers and family members, and they less often chose counselors (Xiaomin and Guangrong, 2004). They only seek professional counseling help when during severe psychological distress (Guangrong and Ming, 2003). Xiangrong and Ying (2014) found that nearly 70% of college students were unfamiliar with campus counseling agencies, and when encountering psychological problems, college students display autonomy and unwillingness to seek help (Xiangrong and Ying, 2014). Xiaomin and Guangrong (2006) studied psychological help-seeking behavior among college students in Wuhan City. Perceived behavioral disorders and self-efficacy were explored in depth; the study revealed that the better a family's economic conditions, the higher the likelihood that individuals would seek psychological counseling when they encountered psychological distress. Additionally, internally-controlled individuals would be more willing to seek help than externally-controlled individuals; counseling self-efficacy has a significant positive correlation with the desire to seek help.

The factors that influence potential clients' mental health help-seeking fall into three broad categories: demographic factors, social, cultural, and psychological. The health belief model (HBM) proposed by I.M. Rosenstock in the 1950s followed the principle of cognitive theory to conceptualize help-seeking behavior and emphasized the dominant role of an individual's subjective psychological processes (i.e., expectations, thinking, reasoning, beliefs, etc.) on behavior in the course of the model's wide application and development. The influence of these psychological processes on health help-seeking behavior reflected in the model have been necessary for several domains and therapeutic work with essential implications (Mantler, 2013; Gu et al., 2014; Conner and Norman, 2015; Huang et al., 2016; Wisutwattanasak et al., 2023).

Undergraduates' psychological problems caused by family, social, and academic reasons have become the social focus of today's era (McArthur et al., 2018; Chen X. et al., 2022). However, most studies have used the HBM in the medical field, and fewer have employed the model to study undergraduates' mental health. This study will address the negative impacts of mental health problems on undergraduates and use the HBM to analyze the factors that promote and hinder undergraduates' mental health help-seeking behavioral intentions. The goal is to intervene in undergraduates' mental health problems effectively and help adolescents to better utilize mental health services.

## 2 Literature review and framework

According to Rosenstock, motivating a person to avoid sub-healthy behaviors requires the individual to satisfy the following conditions (Rosenstock, 1974):

(i) the belief that he is susceptible to a particular disease;
(ii) that the disease will have a more severe impact on his life; and
(iii) that it is beneficial for him to take specific measures against the disease.

Additionally, he must consider subjective judgments about the barriers he could face in adopting healthy behaviors, such as time constraints, financial costs, and the reliability of health care services. The HBM synthesizes motivational, cognitive, and expectancy-value theories to predict whether people can prevent and be aware of a disease and whether they will take countermeasures when indisposed. The model focuses primarily on the relationship between personal health behaviors and personal health, including perceived threat and behavioral assessment (Glanz et al., 2015). The model views perceived threat from two angles: individual perception of their susceptibility to a disease or health problem and the expectation of severe consequences of the disease. Behavioral appraisal focuses on the possible benefits, effects, costs, and barriers of adopting health behaviors. Furthermore, the model suggests that appropriate beliefs are cues to action to motivate individuals to adopt health behaviors. These cues may include various triggers, such as personal perceptions of disease or health behaviors, social influences, health education activities, etc.

Several studies have used health belief models as the classic theoretical model for health behavior research. They usually focus on three main areas: (i) preventive healthcare behaviors, including health-promoting and health-hazardous behaviors, as well as vaccinations and contraceptives (Bryan et al., 1997; Gerend and Shepherd, 2012); (ii) patient role behaviors, which are generally used in studies on adherence to healthcare protocols (Jialie et al., 2020); and (iii) clinical aspects that are used to study patients' motivation to actively seek medical care (Conner and Norman, 2015). For example, scholars have used the HBM to study HPV vaccination (Gerend and Shepherd, 2012), smoking (Mantler, 2013), self-care of diabetic patients (Gillibrand and Stevenson, 2006), health screening behavior studies (Huang et al., 2016), and voluntary premarital medical examination studies (Gu et al., 2014). Similarly, applying the HBM to mental health research in China is not shared. In previous studies, the HBM has mainly been applied to study individuals' perceptions of mental illness and self-treatment behaviors. Meanwhile, it has not received sufficient attention regarding exploring individuals' seeking of mental health services.

In this study, we selected the influential HBM as the primary theoretical model for exploring the potential influences of help-seeking behavior, and the main variables include the following six dimensions: perceived susceptibility, perceived severity, perceived health behavior benefits, perceived barriers, self-efficacy, and cues to action (Rosenstock, 1974).

### 2.1 Perceived susceptibility and behavioral intention

Perceived susceptibility refers to an individual's judgment of the likelihood of suffering from a particular disease or developing a specific health. For example, in a study on condom use by women, the conclusion indicated that women's perceived analysis of STD susceptibility leads to behavioral intention to use condoms (Bryan et al., 1997); Perceived susceptibility refers to an individual's judgment of their likelihood of suffering from a particular disease or developing a specific health challenge. For example, in a study on condom use by women, the conclusion indicated that women's perceived analysis of STD susceptibility leads to their behavioral intention to use condoms (Falck et al., 1995). However, the interpretation of the correlation between perceived susceptibility and health behaviors varies across studies. For example, Adih and Alexander (1999) also examined the determinants of prevention of HIV infection by condom use based on the HBM and found that perceived susceptibility to the virus significantly influenced HIV prevention behaviors. In contrast, a similar study on Asian American college students revealed that perceived susceptibility did not predict HIV prevention behaviors (Yep, 1993).

These two seemingly contradictory results are both practically reasonable. If a person believes he is at risk of contracting HIV and therefore decides to use a condom during sexual intercourse, perceived susceptibility to HIV positively is influencing the individual's behavioral intention. If the same person always uses condoms, perceived susceptibility is low; thus, their perceived susceptibility has no significant effect on behavior (Conner and Norman, 2015). Jorm (2012) hypothesized that a lack of awareness of health problems hinders health-seeking behaviors, and patients who are unaware of mental health problems or certain symptoms that cause illness are less likely to seek help (Goldney et al., 2002). That is, in previous studies, individuals with lower perceived susceptibility were less likely to produce mental health help-seeking behaviors, so we hypothesized that in the case of university students' mental health-seeking behaviors, perceived susceptibility would similarly affect university students' mental health-seeking behavioral intentions. Therefore, we propose the following hypotheses:

H1: Perceived susceptibility positively influences undergraduates' mental health-seeking behavior.

### 2.2 Perceived severity and behavioral intention

Perceived severity, which refers to the degree to which individuals perceive that a disease or unhealthy problem is harmful to them, is the risk factor most widely associated with emotional and behavioral responses and is closely linked to the degree to which an individual's health is and will be threatened (Goldney et al., 2002). Therefore, increasing an individual's perception of the severity of disease symptoms increases the likelihood that they will seek treatment (Henshaw and Freedman-Doan, 2009). As Coulton and Frost studied older people's use of social and health services; they found that more senior people experiencing chronic illness may fail to recognize that they may be suffering from depression or anxiety, because they do not correctly perceive the dangers of mental illness or are unaware of the severe consequences of the condition. Both can lead to the perception that when they are suffering from somatization, they believe that such symptoms are simply the sequelae of a physical illness or a decline in organ functioning due to aging Coulton and Frost (1982). They do not recognize that they may be suffering from depression or anxiety and, therefore, lose a desire for treatment (Smalbrugge et al., 2005). Thus, their willingness to seek treatment significantly reduces.

At the beginning of the influenza A (H1N1) pandemic in Hong Kong, Lau et al. (2010) found that people's perception that influenza A (H1N1) could cause irreversible physical damage or mental distress were significantly associated with handwashing behavior more frequently than 10 times a day. The researchers concluded post-analysis that the higher the perceived severity of the pandemic, the higher the motivation to engage in self-protective behaviors.

In a study of COVID-19 prevention among the Chinese public, Li et al. (2020) found that people who perceived the severity of the virus adopted preventive behaviors to protect themselves against the virus. Similarly, Vogel et al. (2021) found that people with higher perceived severity may practice social distancing and maintain hand hygiene to reduce their likelihood of COVID-19 infection. According to a previous study by Kashyap et al. (2020), smokers may be more likely to be infected with COVID-19 and to develop severe viral complications, found a significant negative association between smoking and increased frequency of smoking in the study population.

A certain level of fear and worry (i.e., prevention is better than cure) can induce desirable preventive behaviors (Lau et al., 2010). Similarly, in the case of illness, people are encouraged to seek treatment because they are aware of the dangers of the disease to them. Undergraduates are also likely to be aware of the harm caused by mental health problems (such as physical health problems, changes in interpersonal relationships, and a decline in academic performance, etc.) and have the will to actively seek a solution. Therefore, we propose the following hypothesis:

H2: Perceived severity positively influences undergraduates' mental health-seeking behavior.

### 2.3 Perceived benefits of health behaviors

Perceived benefits of health behaviors refer to an individual's subjective assessment of the benefits of adopting a particular behavior. It includes whether the individual believes the behavior is beneficial to their health and their expectations of the extent to which the behavior will bring benefits. The correlation between perceived health behavior benefits and health behavior intentions has been affirmed to varying degrees in several studies in different fields that have used the HBM, such as examining the determinants of nurses' intention to vaccinate (Chen et al., 2019), and predicting preventive behaviors for dental caries in students (Oveisi et al., 2019). In response to the hypothetical emergence of the H5N1 epidemic, Lau et al. (2007) studied the preventive behaviors adopted by the Chinese population in Hong Kong. The majority of respondents believed that hand washing was effective in preventing influenza and SARS and, therefore, equally effective in preventing human-to-human transmission of avian flu. Secondly, 90% of the population indicated that they had used masks in public places during the peak of the SARS epidemic (Lau et al., 2003, 2005), and based on such perceptions, the percentage who chose to wear masks in public places was as high as 92.4%.

Unsurprisingly, this is because the population recognized and acknowledged the efficacy of hand washing and mask wearing from their prior experience, with perceptions of their benefits being grounded in preventing SARS and continuing such practices as preventative behaviors against H5N1. Moreover, Gerrard et al. (1996) have called for the identification of mediators influencing the relationship between perceived susceptibility and safer sex behavior. In a subsequent survey of female condom use, Bryan et al. (1997) proposed the benefits perceived by women when using condoms as a mediator between perceived susceptibility and safer sex intentions and concluded in their study that perceived health behavioral benefits mediate the relationship between perceived susceptibility and safer sex.

Concerning research related to mental health-seeking behaviors, psychologists have called for the need to increase public awareness of the benefits of psychotherapy (Policy and Board, 2005). Kim and Zane (2016) found that perceived health behavioral benefits were significantly associated with mental health help-seeking behaviors among Asian American and White American students. Therefore, in addition to speculating that perceived health behavior benefits have a direct impact on undergraduates' mental health help-seeking behaviors, we also suggest that when undergraduates are in a state of high perceived susceptibility to their illnesses, their perceived benefits of addressing their mental illnesses may motivate them to initiate help-seeking. Therefore, we propose the following hypotheses:

H3: Perceived health behavior benefits positively influence undergraduates' mental health help-seeking behavior.
H4: Perceived health behavior benefits mediate the relationship between undergraduates' perceived susceptibility and their mental health help-seeking behavior.

### 2.4 Perceived barriers and behavioral intention

Perceived barriers are individuals' subjective judgments about the roadblocks to adopted health behaviors, such as time spent, financial burden, fear of privacy exposure, and poor service accessibility. Some studies have shown that perceived behavioral barriers are the strongest predictors of health behaviors (Zhu et al., 2003). For example, Oveisi et al. (2019) discovered that students' preventive behaviors, as predicted by the HBM, were among the most important factors influencing the prevention of dental caries. In addition to the healthcare field, psychologists believe that the current focus of mental health is to reduce the stigma associated with psychotherapy (Policy and Board, 2005) and remove barriers that prevent people from engaging in mental health help-seeking behaviors (American Psychological Association, 1992). Orji et al. suggest that perceived barriers remain the only variable negatively affecting health behaviors. Therefore, perceived behavioral barriers can negatively impact undergraduates' mental health help-seeking behaviors (Orji et al., 2012). Therefore, perceived behavioral barriers can negatively impact undergraduates' mental health help-seeking behaviors. Second, it has been noted that the likelihood of an individual taking preventive measures is based on the perceived benefits of the behavior change minus the perceived barriers at the time of the behavior change (Kim et al., 2012; Glanz et al., 2015). In this regard, we believe that changes in perceived health behavioral barriers may affect the interaction term between perceived behavioral benefits and perceived behavioral barriers. Therefore, we proposed the following hypotheses:

H5: Perceived behavioral barriers negatively affect undergraduates' mental health help-seeking behavior.
H6: There is a negative moderating effect of perceived behavioral barriers on the relationship between perceived health behavioral benefits and mental health help-seeking behaviors among undergraduates.

### 2.5 Self-efficacy and behavioral intention

Self-efficacy refers to an individual's evaluation of their ability to control their personal and external factors to adopt health behaviors and achieve desired outcomes successfully. The initial HBM had low predictive validity. Orji et al. (2012) confirmed the predictability of self-efficacy by adding the variable self-efficacy and illustrated that self-efficacy was the most vital determinant in the model. In oral health, self-efficacy has presented a robust predictive role as an essential construct of the HBM (Mehri and Morowatisharifabad, 2009). Oveisi et al. (2019) detected that self-efficacy was a significant predictor of toothbrushing and flossing among dental. Self-efficacy has also been noted as an important factor affecting caries prevention among students in addition to perceived behavioral disorders. In conclusion, self-efficacy would also have a significant impact on mental health help-seeking behavioral intentions among undergraduates. Therefore, we proposed the following hypothesis:

H7: Self-efficacy positively influences undergraduates' mental health help-seeking behavior.

### 2.6 Cues to action and behavioral intention

Cues to action refer to the instructions for action that people receive, including propaganda from media campaigns, reminders from medical personnel, advice from others, and experiences of illnesses from friends and relatives. In a study on the factors influencing the health checkup behaviors of Taiwanese residents, it was found that the influence of doctors' and nurses' advice or media campaigns prompted participants to conduct self-examinations, i.e., cues to action positively influenced residents' behavioral intentions (Huang et al., 2016). Similarly, in a study of breast and colorectal cancer survivors, the results also indicated that cues to action given by healthcare professionals could positively influence secondary cancer prevention behaviors (Baek and Choi, 2023). Furthermore, in similar studies on facilitators of fall prevention in older adults (Vincenzo et al., 2022), and information seeking and motor autonomy in older adults with chronic diseases (Chou and Wister, 2005), the positive effect of cues on action has been confirmed to varying degrees.

While several of the above studies have pointed to cues to action as triggers of health behaviors, a fragile effect of cues to action on health behaviors was found in an extended study of the HBM (Orji et al., 2012). Gerend et al. (2004) argued that cues to action are cues that can directly or indirectly influence a disease's severity (e.g., symptoms, modes of transmission, and its social consequences) by providing information about the individuals' perceptions. Therefore, in the current study, we need to explore which variables are influenced by cues to action. In Mary et al.'s survey of school-aged women, cues to action were found to influence participants' behavioral attitudes by affecting perceived susceptibility. Their data showed a general increase in susceptibility to HPV infection, self-efficacy and willingness to vaccinate among participants who had been advised by their doctors to get the HPV vaccine (Gerend and Shepherd, 2012). Therefore, we chose to explore the relationship between cues to action and perceived susceptibility in addition to speculating the direct effect of cues to action on undergraduates' mental health help-seeking behaviors. Therefore, the following hypotheses were formulated:

H8: Cues to action positively affect the mental health help-seeking behavior of undergraduates.
H9: Cues to action mediate between undergraduates' perceived susceptibility and undergraduates' mental health-seeking behavior.

## 3 Method

### 3.1 Variable measurement and questionnaire design

**Figure 1:**
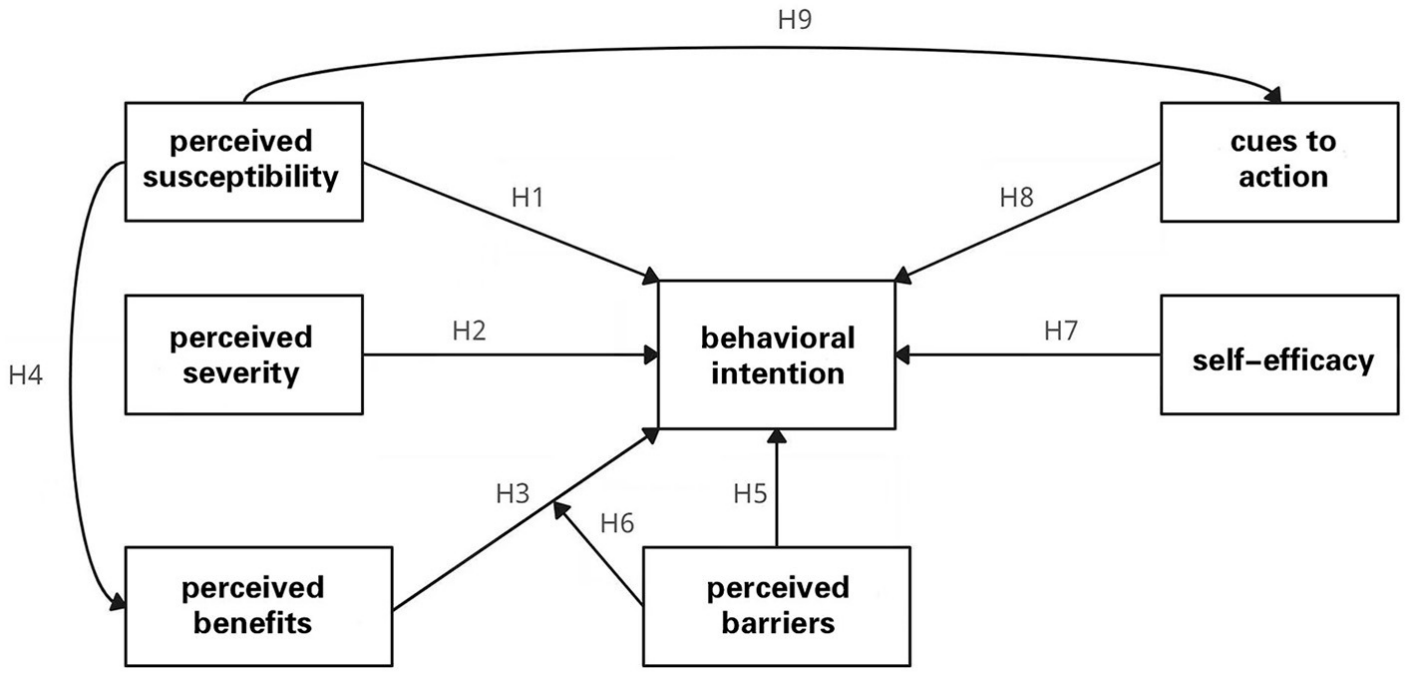
Model of factors influencing mental health help-seeking behavioral intentions.

This study explored the relationship between six factors in the HBM and university students' health help-seeking behaviors (as shown in Figure 1), and the questionnaire contained 47 questions divided into eight dimensions in the following order: (1) perceived susceptibility; (2) perceived severity; (3) perceived health behavioral benefits; (4) perceived behavioral barriers; (5) self-efficacy; (6) cues to action; (7) mental health help-seeking behavioral intentions; and (8) Demographic variables. The questionnaire was scored and quantified using a five-point Likert scale, “Strongly Disagree,” “Disagree,” “Neutral,” “Agree,” and “Strongly Agree” were scored from 1 to 5 in the middle of the two options, and the demographic variables include “gender” (male = 1, female = 2).

### 3.2 Data collection and analysis methods

This study adopted an onlinenaire to collect date, and the content of the questionnaire is shown in Table 1. The Questionnaire.com platform was used to design the online questionnaire (www.wjx.wjx). Social media (e.g., Weibo, WeChat, Xiaohongshu, etc.) was used to get respondents using the snowball sampling method. The recipients were asked to forward the questionnaire link to their friends. This study began on 19 November 2023, and 503 questionnaires were collected during the 20-day survey. After disposing off some questionnaires with insufficient answer time, option repetition rate over 80%, and abnormal sample questionnaires, 446 valid questionnaires remained. Among them, 68.39% were female, and 31.61% were male (Table 2). None of the different genders showed significant differences regarding behavioral intentions (*p* = 0.122, *p* > 0.05). We modified the perceived susceptibility, perceived severity, perceived health behavior benefits, perceived behavioral disorders, and cues to action scales developed by McClenahan et al. (2007). The self-efficacy scale (Cronbach's α= 0.822) was developed by Jayanti and Burns (1998). The behavioral intentions scale (Cronbach's α= 0.862) was developed by Huang et al. (2016). All Cronbach's α values ranged from 0.81 to 0.93 (Table 3).

**Table 1:** Measurement scales and sources.

**Concept**	**Question code**	**Question content source**	**Source**
Perceived			
susceptibility	PSS1	I'm worried about developing mental health related issues	McClenahan et al., 2007
	PSS2	I am at high risk of having mental health problems	
	PSS3	I am more likely than the average person to have mental health problems	
	PSS4	It is highly likely that I will develop mental health problems in the future	
	PSS5	My family history puts me at risk of mental health problems	
	PSS6	I am at high risk of developing mental health problems because of my physical and mental health conditions	
	PSS7	Stress in my life puts me at risk of mental health problems	
Perceived			
severity	PSV1	I am scared to think about mental health problems	McClenahan et al., 2007
	PSV2	The thought of having a mental health problem scares me	
	PSV3	My heart races at the thought that I might have a mental health problem	
	PSV4	I feel anxious at the thought that I might have a mental health problem	
	PSV5	A mental health problem could threaten my relationship with my family or friends	
	PSV6	My financial security would be at risk if I had a mental health problem	
	PSV7	If I have a mental health problem, I will be troubled for a long time	
	PSV8	If I have a mental health problem, my whole life will change	
	PSV9	If I have a mental health problem, my life will be at risk	
Perceived			
benefit	PBN1	If I get help for my mental health problem, it will take a burden off my shoulders	McClenahan et al., 2007
	PBN2	Medication can be an effective treatment for mental health	
	PBN3	Professional therapy can be successful in treating my mental health problems	
	PBN4	Getting help for my mental health problem will make me feel better	
	PBN5	Getting help for my mental health problem will make me feel better with my family and friends	
	PBN6	Getting help for my mental health problem will improve my ability to cope at home, at school, and at work.	
Perceived			
barriers	PBR1	Seeking help for my mental health problem has affected my life	McClenahan et al., 2007
	PBR2	I am worried that I will not be able to get help for my mental health problem from a professional.	
	PBR3	If I go to a specialist or doctor for a mental health problem, they will not understand people like me	
	PBR4	Some people will laugh at me if I seek help for my mental health problems	
	PBR5	Seeking help for a mental health problem takes too much time	
	PBR6	Seeking help for mental health problems costs too much money	
	PBR7	Seeking help for mental health problems is embarrassing	
	PBR8	Seeking help for mental health problems makes me feel miserable	
Self-efficacy	SE1	I feel it is important to do activities to improve my emotional health	Jayanti and Burns, 1998
	SE2	Maintaining good mental health is important to me	
	SE3	I eat a balanced diet	
	SE4	I usually look after my mental health	
	SE5	I have regular mental health checks even when I am not ill	
	SE6	I can recognize emotional problems early	
	SE7	I seek information about mental health to improve my emotional wellbeing	
Cues to action	CTA1	Advice from my doctor or nurse prompts me to seek mental health help	McClenahan et al., 2007
	CTA2	I am prompted to seek mental health help by a family member or friend who is ill.	
	CTA3	Activities (e.g., media: press, TV, radio, internet) may prompt me to seek mental health help.	
	CTA4	Symptoms of mental illness (e.g., somatization) motivate me to seek mental health help	
	CTA5	Experiences related to mental health problems that prompt me to seek mental health help	
behavioral			
intention	BI1	Each month in the future, I will look at my mental health and seek help for it.	Huang et al., 2016 [-0.7pt]
	BI2	In the coming month, I will try to seek help for my mental health.	
	BI3	In the coming month, I intend to seek help for my mental health.	

**Table 2:** Results of descriptive statistical analysis of the formal questionnaire (*N* = 446).

**Item**	***N***	**%**
Gender		
Male	141	31.61
Female	305	68.39

**Table 3:** Internal consistency, convergent validity analysis.

**Variant**	**Cronbach's α**	**CR**	**AVE**

Perceived susceptibility	0.926	0.928	0.651
Perceived severity	0.926	0.926	0.583
Perceived behavioral benefits	0.873	0.877	0.552
Perceived behavioral difficulties	0.874	0.876	0.476
Self-efficacy	0.814	0.823	0.402
Cues to action	0.868	0.868	0.569
Behavioral intentions	0.859	0.872	0.697

### 3.3 Internal consistency, convergent and discriminant validity

The Cronbach's α coefficient was used to determine the consistency of variables within each dimension. In contrast, the validated factor analysis was used to test the convergent validity and discriminant validity of each dimension (Cortina, 1993), whose evaluation indexes generally included standardized factor loading, average variance extracted (AVE), and composite reliability (CR).

### 3.4 Statistical analysis

This study used structural equation modeling (SEM) to validate the model and hypotheses. SEM is a statistical method, which enables an argumentative analysis of the structural theory of a particular phenomenon (Bentler, 1988). The data analysis followed Anderson and Gerbing's methodology (Anderson and Gerbing, 1988). The measurement model consisting of the seven factors in the study was first assessed in terms of reliability, convergent validity, and discriminant validity of the scale. Then, the model consisting of the seven variables was individually validated.

## 4 Results

### 4.1 Reliability and validity test

The data were analyzed using IBM SPSS statistical software. Firstly, the reliability of the factors in the scale was analyzed (Table 3). The results showed that the Cronbach's alpha values of all the required measures exceeded 0.8, which proved that the scales were highly reliable. The potential variables measured were internally consistent (Cortina, 1993). Next, we used validated a factor analysis to test the convergent validity and composite reliabilities and AVEs of the variables to measure the convergent validity of the variables and observed variables. The composite validity of all dimensions in this study exceeded 0.7, indicating that all dimensions had good convergent validity. Finally, all values in this study met the criterion for discriminant validity (e.g., 0.807 > 0.653), which indicates that the dimensions in this study have good discriminant validity (Table 4).

**Table 4:** Distinguishing validity: Pearson's correlation and AVE square root values.

	**1**	**2**	**3**		**5**	**6**	**7**
Perceived susceptibility	0.807						
Perceived severity	0.653	0.763					
Perceived behavioral benefits	0.091	0.208	0.743				
Perceived behavioral difficulties	–0.487	0.65	–0.104	0.69			
Self-efficacy	–0.194	–0.041	0.417	0.14	0.634		
Cues to action	0.166	0.258	0.321	–0.199	0.465	0.755	
Behavioral intentions	0.142^**^	0.226^**^	0.203^**^	–0.152^**^	0.388	0.481^***^	0.835^**^

^**^p < 0.01.

^***^p < 0.001.

### 4.2 Structural model results

We utilized AMOS software (IBM software, Armonk, NY, USA) for structural model analysis. The GFI obtained was 0.698, RMSEA was 0.076, PGFI was 0.623, PCFI was 0.764, and SRMR was 0.081. Thus, the study's findings suggest that the theoretical model fits well.

### 4.3 Hypothesis testing

#### 4.3.1 Regression analysis test

**Figure 2:**
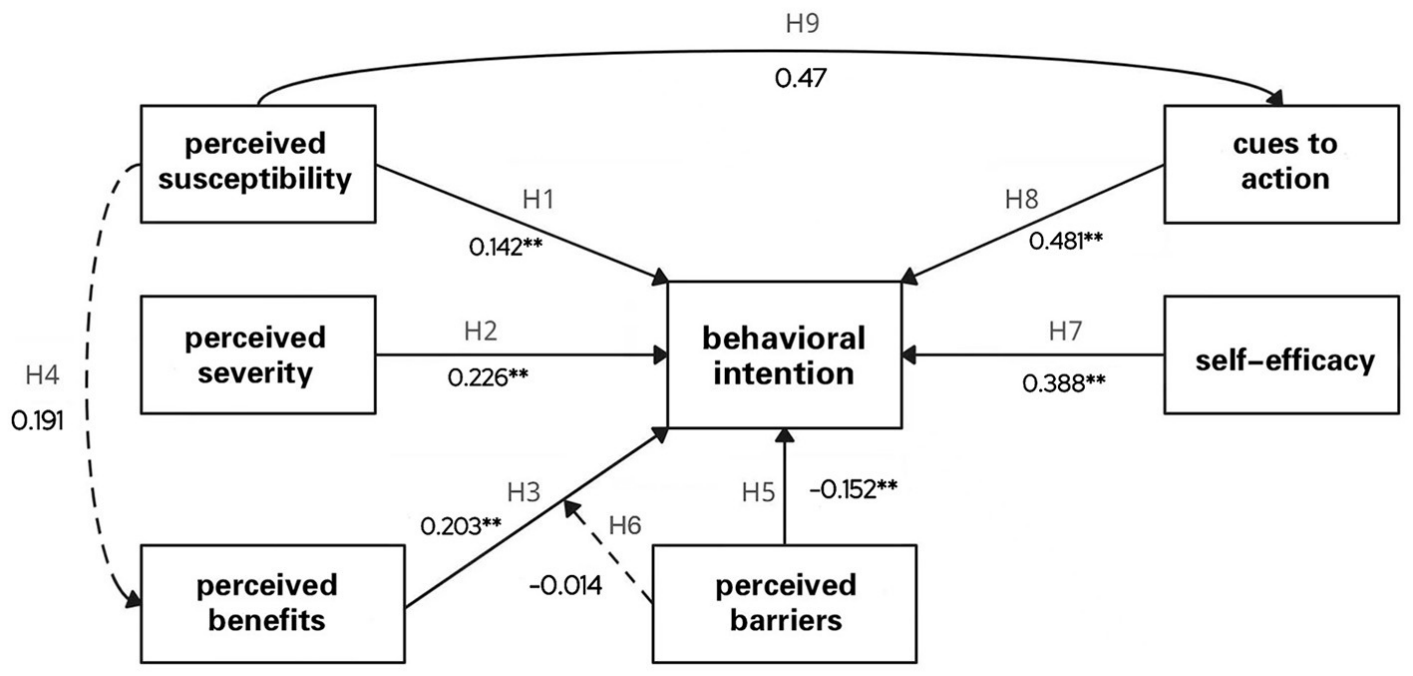
Research model path coefficients.

Figure 2 demonstrates the relationship between variables, where ** denotes *p* < 0.01. The standardized coefficient of perceived susceptibility was 0.142, with a *p*-value of < 0.01, indicating that this relationship is statistically significant, supporting H1. The standardized coefficient of perceived severity was 0.226 stronger than perceived susceptibility. It implies that perceived severity has a more significant effect on related behavioral intentions. Again, a *p*-value of < 0.01 indicates that this relationship is statistically significant, supporting H2. The standardized coefficient of 0.203 for perceived behavioral benefits also positively affects related behavioral intentions. A *p*-value of < 0.01 indicates that this relationship is statistically significant and supports H3. The standardized coefficient for perceived behavioral difficulties is –0.152, which means that perceived behavioral difficulties was negatively related to related behavioral intentions. Ap-value of < 0.01 indicates that this relationship is statistically significant and supports H5. The standardized coefficient of self-efficacy was 0.388, and the *p*-value was < 0.01, indicating that this relationship is statistically significant and supports H7. The standardized coefficient of cues to action was 0.481, which is the highest among all the independent variables, indicating that the effect of cues to action on related behavioral intention was highly significant, and a *p*-value < 0.01 indicates that this relationship is statistically significant and supports H8. In summary, based on the results, H1, H2, H3, H5, H7, and H8 were supported, i.e., there is a significant association between perceived susceptibility, perceived severity, perceived behavioral benefits, perceived behavioral disorders, self-efficacy, and cues to action and behavioral intention.

We noticed a significant association between cues to action and behavioral intention, which counters previous overseas studies. In order to further comprehend the role of cues to action, this variable was analyzed in detail by the researcher. In conclusion, except for the two items “I am prompted to seek mental health help by a family member or friend who is ill” and “Symptoms of mental illness (e.g., somatization) motivate me to seek mental health help,” the other three items have a significant effect on participants' behavioral intention, i.e. professional advice (advice from doctors and nurses) had a more profound effect on participants' behavioral intention than family and friends and participants' own feelings.

#### 4.3.2 Mediating effect test

Perceived susceptibility positively influences undergraduates' mental health-seeking behavior. To further understand the relationship between perceived susceptibility and undergraduates' cognitive health help-seeking behavior, this study examined whether perceived susceptibility can affect undergraduates' mental health help-seeking behavior under specific conditions.

Firstly, the study explored H4—whether the relationship between perceived susceptibility and behavioral intentions is more significant in contexts of high perceived behavioral benefit. This hypothesis aimed to investigate whether groups who perceive themselves to be more likely to develop a mental illness would be more likely to seek help and whether behavioral intentions would be stronger if they were able to recognize the perceived benefits of mental health services. Secondly, the study explored H9—whether there is a possibility that the relationship between perceived susceptibility and behavioral intention becomes more significant with more precise cues to action; specifically, whether individuals are more likely to initiate help-seeking when they perceive that they are at risk of developing a mental illness or when inspired or advised by others to do so.

We concluded from the regression analysis that perceived susceptibility significantly and positively predicted undergraduates' mental health help-seeking behavioral intentions, perceived behavioral benefits significantly and positively predicted undergraduates' mental health help-seeking behavioral intentions, and cues to action significantly and positively predicted undergraduates' mental health help-seeking behavioral intentions. We standardized all the variables from Wen et al. (2005). After controlling for variables, such as gender, we tested the mediating roles of perceived behavioral benefits and cues to action in the relationship between perceived susceptibility and undergraduates' mental health help-seeking behavioral intention. Here, we used the bias-corrected Bootstrap method to test the significance of the mediating effect, and after testing H4 the results showed that the mediating effect value was 0.017, the SE value was 0.011, and the 95% CI was (–0.000, 0.045). Thus, the mediating effect of perceived health behavioral benefits between undergraduates' perceived susceptibility and their mental health help-seeking behaviors was not significant, proving that even if people with high susceptibility recognize that mental health services are beneficial to them, their behavioral intentions would not change significantly. The test results of H9 show a mediation effect value of 0.077, with an SE value of 0.027, and a 95% CI of (0.028, 0.133), suggesting that the mediation effect of cues to action between undergraduates' perceived susceptibility and mental health help-seeking behavior was utterly significant. It demonstrates that individuals are more motivated to seek mental health help if they highly perceive that they are at risk of developing a mental illness and are encouraged or counseled by others to seek help.

#### 4.3.3 Moderating effects test

The effect of perceived behavioral benefits on undergraduates' mental health help-seeking behavior and whether perceived behavioral barriers moderate the direct effect was first tested. In Section 4.3.1, perceived behavioral benefits significantly (β = 0.203, *p* > 0.001) positively predicted undergraduates' mental health help-seeking behaviors. In contrast, in the moderated effects test, the interaction term between perceived behavioral benefits and perceived behavioral barriers did not have a significant predictive effect on undergraduates' mental health help-seeking behaviors (β = –0.014, *p* > 0.05), proving that undergraduates' knowledge of seeking mental health services is beneficial to them. Furthermore, it demonstrates that undergraduates are not significantly affected by barriers such as time and money.

#### 4.3.4 Multiple regression analysis

We tested the hypotheses (H1, H2, H3, H5, H7, and H8) for each of the correlations through multiple regression analyses, which yielded a summary of the model regression coefficients (Table 5): perceived susceptibility (*Z* = 1.195, *p* = 0.232 > 0.05), perceived severity (*Z* = 1.555, *p* = 0.120 > 0.05), perceived behavioral benefits (*Z* = –1.040, *p* = 0.298 > 0.05), and perceived health behavior disorder (*Z* = –0.697, *p* = 0.486 > 0.05). None of the four factors showed a significant influence on behavioral intention. Thus, the four variables, namely perceived susceptibility, perceived severity, perceived behavioral benefits, and perceived behavioral disorder, are not independent influencing factors.

**Table 5:** Summary of model regression (path) coefficients.

**Research hypothesis**	**Trails**	**SE**	***Z* (CR)**	**Standardized path factor**	**Test results**
H1	Perceived susceptibility → behavioral intention	0.054	1.195	0.065	Untenable
H2	Perceived severity → behavioral intention	0.066	1.555	0.097	Untenable
H3	Perceived behavioral benefit → behavioral intention	0.059	–1.040	–0.047	Untenable
H5	Perceived behavioral disorders → behavioral intention	0.067	–0.697	–0.037	Untenable
H7	Self-efficacy → behavioral intention	0.077	5.425	0.279^**^	Tenable
H8	Cues to action → behavioral intention	0.056	6.673	0.323^**^	Tenable

→ indicates path influence relationship.

^**^p < 0.01.

Regarding self-efficacy's influence on behavioral intention, we found that the standardized path coefficient value is 0.279 > 0, and this path is significant at the 0.01 level (*Z* = 5.425, *p* < 0.01), which indicates that self-efficacy has a significant favorable influence on behavioral intention. In addition, the path of cues to action on behavioral intention. Additionally, in the path of cues to action on behavioral intention, we found that its standardized path coefficient value is 0.323 > 0, and this path showed significance at the 0.01 level (*Z* = 6.673, *p* < 0.01), thus indicating that cues to action also has a significant favorable influence on behavioral intention.

## 5 Conclusion and discussion

Recently, there has been a growing awareness of the importance of mental health care. According to the World Health Organization, one in seven of the world's 10–19-year-old suffers from a mental disorder, accounting for 13% of the global burden of disease in this age group. Suicide ranks as the fourth leading cause of death for individuals aged 15–29. If mental health issues among undergraduates are not addressed, the adverse effects of the disorders will carry over into adulthood, damaging the physical and psychological health of undergraduates.

As future leaders and contributors to society, undergraduates' mental health directly impacts society's long-term wellbeing. Undergraduates' mental health problems may be exacerbated by academic pressures, social challenges, and career planning issues, affecting their daily lives and academic performance. However, it may also have a profound impact on their mental development. Given the insidious and sudden nature of psychological problems, it is crucial to take proactive preventive measures to reduce the risk of mental illness.

The HBM provides a valuable framework for understanding and preventing psychological problems. Through a series of content extensions, the model explores the impact on undergraduates' mental health help-seeking behaviors from six dimensions; the inclusion of the mediating and moderating effect of cues to action on perceived susceptibility and behavioral intention emphasizes the importance of the intervention pathway of psychological crisis. According to the WHO report, providing mental health support services in schools is the most efficient strategy to improve students' mental health (World Health Organization, 2022). Therefore, preventing undergraduates' psychological crises is crucial to college education and social stability. Establishing and improving the mental health education system and providing professional mental health counseling services in colleges and universities are indispensable interventions.

Practically, this study presents empirical data on the factors influencing the mental health help-seeking behaviors of contemporary Chinese undergraduates. Seven out of the nine hypotheses we put forward were confirmed, and the main conclusions are as follows:

First, perceived susceptibility, perceived severity, perceived behavioral benefits, self-efficacy, and cues to action significantly positively predicted undergraduates' mental health help-seeking behaviors, and perceived behavioral disorders significantly negatively predicted undergraduates' cognitive health help-seeking behaviors. The six factors had a significant correlation with behavioral intention. This finding can be corroborated with previous studies. Second, regarding H9, we learned that the perceived susceptibility of Chinese undergraduates mediates the relationship between cues to action and undergraduates' mental health help-seeking behaviors. Specifically, when undergraduates perceive a particular risk of illness, media publicity, reminders from medical personnel, advice from others, and experience of illness from friends and relatives can promote their mental health help-seeking behavior.

However, in addition to the correlations we tested, we explored this further through multilayer regression analysis. We found that only two factors, self-efficacy, and cues to action, had an independent effect on behavioral intention. It is similar to the findings of O'connor et al. (2014), who concluded that perceived susceptibility, perceived severity, and behavioral cues were not significant predictors of undergraduates' mental health-seeking behaviors which simultaneously exemplifies the limitations of the current study.

Orji et al. have suggested limitations of the HBM, arguing that the predictive power of the model's determinants is low. The model's range of influence is small, confirming the predictive utility of self-efficacy in the model and suggesting that self-efficacy is the most vital determinant in the model (Orji et al., 2012). The focus of the current study was Chinese undergraduates. Self-efficacy and cues to action were significant predictors of undergraduates' mental health-seeking behavior. The predictive validity of self-efficacy was affirmed compared to previous foreign researchers, but the conclusions about the predictive validity of cues to action differed. This result may be due to the difference in the education system of China compared to foreign countries, which we will not discuss here. A significant finding of this study is that Chinese undergraduates believe they are motivated to seek mental health help because they are confident that their mental health problems can be successfully treated and influenced by others' advice, including healthcare professionals, family, and friends, among others. Thus, Chinese undergraduates gain confidence internally and receive cues from others externally. Moreover, different sources of cues to action yield different results. Research has revealed that Chinese undergraduates are more likely to follow professionals' advice, but their own mental illness symptoms (e.g., somatization symptoms) do not significantly influence their behavioral intentions, which is similar to the findings of a local Chinese meta-study. The study indicated that somatization symptoms did not moderate the relationship between problem-solving and psychological wellbeing. Similarly, a local Chinese meta-study indicated that somatization symptoms did not moderate the relationship between problem-solving and mental health. This finding may be related to the level of knowledge of Chinese undergraduates about the symptoms of psychological disorders; this could be explored as a separate research topic (Table 6).

**Table 6:** Summary of model regression (path) coefficients.

**Trails**	**SE**	***Z* (CR)**	**Standardized path factor**	**Test results**
CAT1 → behavioral intention	0.057	3.900	0.243^***^	Tenable
CAT2 → behavioral intention	0.057	–0.331	–0.021	Untenable
CAT3 → behavioral intention	0.051	3.602	0.189^***^	Tenable
CAT4 → behavioral intention	0.058	–0.334	–0.020	Untenable
CAT5 → behavioral intention	0.061	3.315	0.209^***^	Tenable

→ indicates path influence relationship.

^***^p < 0.001.

When the content of persuasive messages are cognitively processed, individual's health perceptions, attitudes, and beliefs could be influenced (Morgan et al., 2010). On this basis, we conducted an in-depth investigation of the relationship between cues to action and mental health-seeking behavior and utilized a moderated effects tests to examine the relationship between other variables in the HBM (perceived behavioral disorders, perceived behavioral benefits, perceived severity, perceived susceptibility, and self-efficacy) and the interaction term of behavioral cues was a significant predict or of mental health help-seeking behaviors among undergraduates. The specific hypotheses were:

H10: A positive moderating effect of perceived susceptibility between cues to action and mental health help-seeking behavior exists.
H11: A positive moderating effect of perceived behavioral benefits between cues to action and mental health help-seeking behavior exists.
H12: A positive moderating effect of perceived severity exists between cues to action and mental health help-seeking behavior.
H13: Self-efficacy has a positive moderating effect between cues to action and mental health help-seeking behavior.
H14: There will be a negative moderating effect of perceived conduct disorder between cues to action and mental health help-seeking behavior.

After data analysis, the results showed that H11 and H14 were partially validated: perceived behavioral benefits had a significant positive moderating effect on the pathway between cues to action and mental health help-seeking behaviors (β= –0.122, *p* < 0.05), and perceived behavioral barriers had a significant negative moderating effect on the pathway between cues to action and mental health help-seeking behaviors (β = –0.088, *p* < 0.05). The more cues an individual receives from others, the more benefits from seeking psychotherapy they perceive and the more motivated they are to seek mental health help actively. Barriers perceived from seeking psychotherapy would hinder willingness to seek help. Therefore, the present study suggests that what Chinese undergraduates need when obtaining mental health advice are increasing cues of benefits. Thus, it behooves on universities to publicly introduce the benefits of counseling and limit mentioning the financial or time pressures students will face during their studies. Additionally, although health literacy (β = –0.37, *p* > 0.05) was not a significant predictor of adolescent mental health help-seeking behaviors (O'connor et al., 2014), we cannot conclude based on the differences between Chinese and foreign countries, as reflected in the cues to action mentioned. No hypothesis regarding health literacy was tested in the current study.

Other limitations of this study arose. This study used a web-based questionnaire to collect sample data in snowball sampling, which limits the generalizability of the findings and may lead to over-representation or neglect of certain groups. In the future, a more scientific sampling method can be used to conduct the study by considering elements such as gender, grade, education, region, etc. We hope to be able to use Respondent Driven Sampling (RDS) analyses and in-depth interviews in the future to compensate for the shortcomings of this paper.

The HBM also poses a limitation to this study. Even though in the subsequent versions of the model, researchers have improved the HBM by adding new measurement factors (e.g., health motivation included by Conner and Norman, 2015), the influence of individual emotions and their context on health-seeking behaviors can still not be fully considered due to their limited prediction accuracy. Subsequently, researchers can improve the predictive accuracy by adding other more valid measures and conducting face-to-face offline interviews to fully understand the potential factors influencing respondents' behaviors.

Finally, in this study, we failed to demonstrate that behavioral disorders have a significant moderating effect between perceived behavioral benefits and undergraduates' mental health-seeking behaviors, and other hypotheses have not been proposed to be explored. Future research should address the moderating effect between variable factors to compensate for the missing relationship between the predictors.

## Data availability statement

The original contributions presented in the study are included in the article/supplementary material, further inquiries can be directed to the corresponding author.

## Author contributions

HW: Conceptualization, Methodology, Writing—original draft. ZF: Conceptualization, Project administration, Writing—review & editing. ZZ: Investigation, Resources, Writing—original draft. JY: Data curation, Investigation, Writing—original draft.
